# What is your diagnosis?

**DOI:** 10.4274/jtgga.2016.0220

**Published:** 2017-03-01

**Authors:** Mehmet Özsürmeli, Selim Büyükkurt, Önder Özden, Mete Sucu, Cihan Çetin, Fatma Tuncay Özgünen

**Affiliations:** 1 Department of Obstetrics and Gynecology, University of Çukurova, School of Medicine, Adana, Turkey; 2 Department of Pediatric Surgery, University of Çukurova, School of Medicine, Adana, Turkey; 3 Department of Obstetrics and Gynecology, Derince Training and Research Hospital, Kocaeli, Turkey

A gravid 2 para 1 woman aged 20 years, presented at the 35^th^ week of her pregnancy. Her medical history was unremarkable. Prenatal evaluation was performed using a Voluson E6 with a convex volumetric transducer (RAB 6-D 2-7 MHz) probe (GE, Zipf, Austria). During an ultrasound evaluation of the fetus, a hyperechogenic mass was detected in the right adrenal gland region. The mass was 30x26x15 mm in size (Figure 1). Doppler investigation showed no vascularization in the mass. Middle cerebral artery peak systolic velocimetry measurement did not show fetal anemia. Fetal anatomic structures, echocardiography, and biometry were otherwise normal. The mass showed a minimal decrease in size in subsequent ultrasound examinations; however, the center of the mass gradually became hypoechogenic and cystic over time. What is your diagnosis?

## ANSWER

At 40 weeks of gestation, the woman delivered a male infant vaginally. Postnatal ultrasonographic evaluation showed a mass on the right adrenal gland. The mass was confirmed to be an adrenal hematoma during serial ultrasonographic evaluations. The newborn was otherwise noted to be healthy. The lesion spontaneously and completely regressed at the fourth month of life. During this time, no complications occurred. The newborn had no thrombocytopenia, anemia or any endocrine disorders. Adrenal gland hormone levels were normal.

The incidence of adrenal hemorrhage has been estimated to be 1.9/1000 in live births (1). The causes of fetal adrenal hemorrhage are poorly understood. The pathophysiology could be related to a sudden increase in intravascular pressure. The fetal adrenal glands are responsive to trauma and hemorrhage because of their relatively large size and vascularization (2). It has been described in association with fetal renal vein thrombosis, Beckwith-Wiedemann syndrome, and Galen vein aneurysm (3, 4). The right adrenal gland is involved in 75% of cases; the reason of this is likely due to the relatively shorter adrenal vein (5).

Prenatal ultrasound should be able to differentiate abdominal masses from adrenal masses. The differential diagnoses of fetal adrenal masses include neuroblastoma, extra lobular pulmonary sequestration, bronchogenic cysts, and adrenal cysts other than adrenal hemorrhage (6, 7). If hemorrhage becomes more complex sonographically, differentiation from neuroblastoma may be difficult. This is due to the fact that they have many similar ultrasonographic findings and also because adrenal hemorrhage could be a complication of neuroblastoma. Color flow Doppler evaluation can show blood flow in a neuroblastoma, which is always absent in a hematoma (5). A combination of ultrasound and magnetic resonance imaging (MRI) gives more definitive information to allow correct diagnosis (8). Bilateral adrenal lesions are most likely adrenal hemorrhage because bilateral adrenal tumors are very rare. In our case, we did not consider MRI because of the advanced gestational age.

The key in the diagnosis of hemorrhage is its change in sonographic appearance over time. At first, it is a solid and echogenic lesion, and then gradually the center of lesion becomes hypoechogenic and cystic, respectively. Finally, the whole lesion decreases in size and looks anechogenic. Dystrophic calcifications may also occur at the final stage (5). Most hemorrhages are reported to occur at birth or during the early neonatal period. Despite these ultrasonographic findings, the prenatal detection rate is much lower than the postnatal life (9). This is probably because adrenal glands are often omitted during prenatal evaluation. Postnatal hormone levels should be evaluated because adrenal hemorrhage may affect adrenal hormone activities (10).

When adrenal hematomas are diagnosed prenatally, patients should be counseled that these lesions can regress spontaneously and regression can even begin prenatally. Vaginal delivery is not contraindicated in these patients. However, potential complications of this situation should also be kept in mind and these patients should be encouraged to deliver in a tertiary healthcare center.

## Figures and Tables

**Figure 1:**
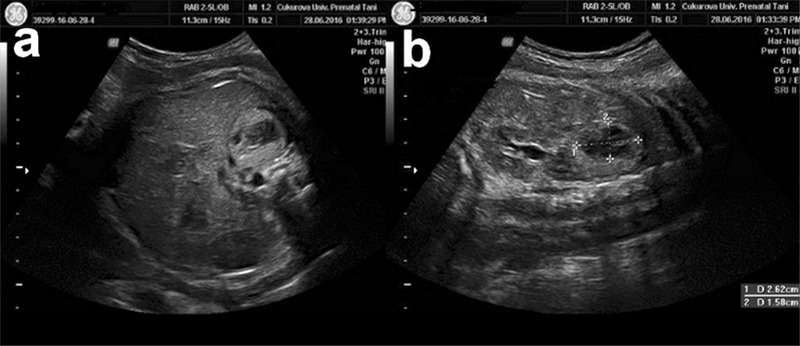
a, b: Axial (a) and coronal (b) view of fetal right adrenal gland is depicted. Central necrosis and hyperechogenic contour signifies bleeding into the surrenal tissue. 67x29 mm
